# Epileptic seizure detection from EEG signals using logistic model trees

**DOI:** 10.1007/s40708-015-0030-2

**Published:** 2016-01-21

**Authors:** Enamul Kabir, Siuly, Yanchun Zhang

**Affiliations:** 1 School of Agricultural, Computational and Environmental Sciences, University of Southern Queensland, Toowoomba, QLD Australia; 2 Centre for Applied Informatics, College of Engineering and Science, Victoria University, Melbourne, Australia

**Keywords:** Electroencephalogram (EEG), Epileptic seizure, Optimum allocation technique (OAT), Logistic model trees (LMT), Classification, Feature extraction

## Abstract

Reliable analysis of electroencephalogram (EEG) signals is crucial that could lead the way to correct diagnostic and therapeutic methods for the treatment of patients with neurological abnormalities, especially epilepsy. This paper presents a novel analysis system for detecting epileptic seizure from EEG signals, which uses statistical features based on optimum allocation technique (OAT) with logistic model trees (LMT). The analysis involves applying the OAT to select representative EEG signals that reflect the entire database. Then, some statistical features are extracted from these EEG signals and the obtained feature set is fed into the LMT classification model to detect epileptic seizure. To test the consistency of the proposed method, all experiments are carried out on a benchmark EEG dataset and repeated twenty times with the same parameters in the detection process, and the average values of the performance parameters are reported. The results show very high detection performances for each class, and also confirm the consistency of the proposed method in the repeating process. The proposed method outperforms some state-of-the-art methods of epileptic EEG signal detection using the same EEG dataset.

## Introduction

Nowadays, the detection of EEG signals is an important key issue in biomedical research for diagnosis and evaluation. The design of multiclass electroencephalogram (EEG) signal detection is a very challenging task because of the need to extract representative patterns from multidimensional time series generated from EEG measurements [1]. Efficiently detecting epileptic seizure EEG signals is beneficial for handling neurological abnormalities and also for evaluating the physiological state of the brain for a broad range of applications in biomedical community. EEG signals indicate the electrical activity of the brain and contain useful information about the brain state to study brain function [2]. The identification of different category EEG signals is traditionally performed by experts based on the visual interpretation. The manual scoring is subject to human errors and it is time consuming, costly process, and not sufficient enough for reliable information [3, 4]. Thus, there is an ever-increasing need for developing automatic systems to evaluate and diagnose of epileptic seizure EEG signals to prevent the possibility of the analyst missing information.

In order to perform the detection of signal’s category, first the most important task is to extract distinguishing features or characteristics from EEG data that can describe the morphologies or the key properties of the signals [5]. The features significantly affect the accuracy of detecting EEG signals [6]. The features characterizing the original EEG are used as the input of a classifier to differentiate different categories of EEGs.

Observing these challenges, this paper looks at the idea of the sampling for getting representative information from raw EEG data for the detection of epileptic seizure EEG signals. A structure is developed for the detection of epileptic seizure EEG signals based on sampling for the feature extraction stage by proposing optimum allocation sampling technique (OAT). A detailed descriptions of the OAT are discussed in Sect. 3.1. The proposed approach consists of the following steps:

1. the whole EEG signals of a class (a category) are divided into several groups according to a particular time period;
2. a representative sample from each group of a class is drawn using the OAT. The ‘OAT’ set is then developed by combining all of the samples from each group of that class;
3. a set of descriptive features are extracted from the OAT set of that class;
4. the same procedure is applied for all of the classes of EEG data. The accumulation of all features from all of the classes constitutes a feature vector for the OAT scheme. The collection of all features from all class of EEG signals for the OAT scheme is employed as an input set in the classifier.

In order to find out an effective model with the highest accuracy for detection of multi-category EEG signals, in this paper, we test an effective machine learning techniques, namely, logistic models trees (LMT) on the composite features. To evaluate the performance of the classifiers, we apply cross-validation procedure to create training and testing sets. The proposed approach is also used to other two well-known classifiers, namely multinomial logistic regression with ridge estimator (MLR) and support vector machine (SVM) on the same competitive features. The experimental results show that the proposed algorithms can detect reasonably for each class of EEG signals by using the LRT classifier.

The rest of the paper is organized as follows. We present a brief overview of multiclass EEG classification techniques in Sect. 2. Section 3 presents a description of the proposed methodology in details. In this section, we also briefly describe the three classifiers and the features extraction methods used in this paper. The description of benchmark EEG data and experimental design is provided in Sect. 4. In Section 5, we present the experimental results of the three classifiers with a detailed discussion. Finally, concluding remarks are included in Sect. 6.

## Related work

This work is related to several multiclass EEG signal classifications techniques in the literature. Siuly and Li [1] proposed a statistical framework for multiclass EEG signal classifications. They developed an optimum allocation scheme based on the variability of observation within a group (based on specific time) of the EEG data and selected a representative sample. The representatives were fed to the least square SVM (LS-SVM) classifier instead of taking representative features that may be a limit for further consideration of a detection technique. Later, Siuly et al. [5] proposed a sampling-based approach for the classification of multi-category EEG signals. The work presented in this paper is similar to them but we use a logistic model trees (LMT) instead of k-nearest neighbour (k-NN). An approach based on a cascade of wavelet-approximate entropy was introduced by Shen et al. [7] for the feature extraction in the EEG signal classification. They tested three existing methods, SVM, *k*-nearest neighbour (*k*-NN), and radial basis function neural network (RBFNN), and determined the classifier of best performance. Acharjee and Shahnaz [8] had a study on twelve Cohen class kernel functions to transform EEG data in order to facilitate the time frequency analysis. The transformed data formulated a feature vector consisting of modular energy and modular entropy, and the feature vector was fed to an artificial neural network (ANN) classifier. Murugavel et al. [9] had conducted a study based on Lyapunov feature and a multiclass SVM for the detection of EEG signals. Ubeyli [10] presented an approach that integrated automatic diagnOATic systems with spectral analysis techniques for EEG signal classification. The wavelet coefficients and power spectral density (PSD) values obtained by eigenvector methods were used as features, and these features were fed to each of the seven classification algorithms (SVM, recurrent neural networks (RNN), PNN, mixture of experts (ME), modified mixture of experts (MME), combined neural networks (CNN), and multilayer perceptron neural network (MLPNN)). Ubeyli [11] provided another algorithm based on eigenvector methods and multiclass SVMs with the ECOC for the classification of EEG signals. In the feature extraction stage, three eigenvector methods such as the Pisarenko, music, and minimum norm were used to obtain the PSD values of the EEG signals that were employed as the input of the multiclass SVMs. For the detection of multiclass EEG signals, Guler and Ubeyli [12] had examined again SVM, PNN, and MLPNN on wavelet coefficients and Lyapunov exponents features.

## Methodology

**Fig. 1:**
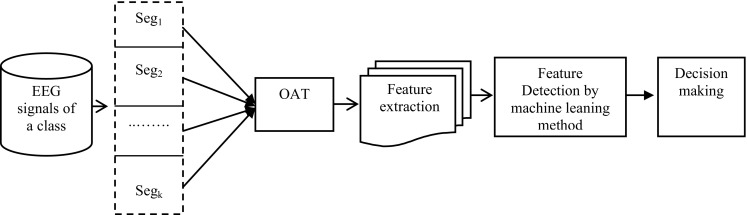
Scheme of the proposed method for the detection of epileptic seizure signals

The proposed technique presented in this paper is depicted in Fig. 1 and is composed of the following three steps:

1. First, divide the whole EEG signal of a class (e.g. healthy, seizure-free, and seizure) into several segments based on specific time interval and then select representative samples by using OAT from each and every segment of the entire signal data of that category. The reason of segmentation is to properly account for possible stationeries as signal processing methods require stationary of signals, while EEG signals are non-stationary, aperiodic, and the magnitudes of the signals are changed over time. The time period is determined viewing the signals periodic patterns in each class within a time casement. As can be seen in Fig. 1, in this study, the EEG signals of each class is divided into *k* non-overlapping segments denoted as Seg_1_, Seg_2_,…,Seg_*k*_ considering a particular time period. Then, the representative observations are selected from each segment by the OAT.
2. Extract representative features from the OAT segments are to represent the distribution of data pattern and then to integrate all of the features of each class in a matrix, called feature vector set.
3. Use three different machine learning detection techniques, namely LMT, MLR, and SVM for the detection of epileptic seizure EEG signals as shown in Fig. 1.

### Optimum allocation sampling technique (OAT)

In this scenario, we firstly determine the required sample size from the whole EEG signals of a particular class with a desired confidence interval and confidence level. The required sample size of the whole data of a class is determined by using Eqs. (1) and (2) [1].

1 $$n_{0} = \frac{{z^{2} \times p \times (1 - p)}}{{d^{2} }},$$

where $n_{0}$ is the sample size; *z* is the standard normal variate (*Z*-value) for the desired confidence level; *p* is the assumed proportion in the target population estimated to have a particular characteristic; and *d* is the margin of error or the desired level of precision. If the number of observation in a particular class is known as *N*, we use the following formula to determine the sample size (*n*) in each class.

2 $$n = \frac{{n_{0} }}{{1 + \frac{{n_{0} - 1}}{N}}}$$

Once we determined the sample size (*n*), the next step is to determine the number of sample from each and every segment. We use OAT scheme in order to determine the required sample from each segment using Eq. (3) that considers the variability among the signals in each segment. A detail description of the OAT is available in references [1, 5].

3 $$n(i) = \frac{{N_{i} \sqrt {\sum\nolimits_{j - 1}^{p} {S_{ij}^{2} } } }}{{\sum\nolimits_{i - 1}^{k} {\left( {N_{i} \sqrt {\sum\nolimits_{j - 1}^{p} {S_{ij}^{2} } } } \right)} }} \times n\;\;\;\;\;\;i = 1,2, \ldots ,k;\;j = 1,2, \ldots ,p,$$

where $n(i)$ is the required sample size of the *i*th Seg; $N_{i}$ is the data size of the *i*th Seg; $s_{ij}^{2}$ is the variance of the *j*th channel of the *i*th Seg; and *n* is the sample size of the EEG recording of a class obtained. Then all of the selected samples from the segments of each class are united in a set (named OAT set) and representative characteristics are calculated from the OAT set as discussed in Sect. 3.3.

### Feature extraction

The feature extraction process transforms the original signals into a feature vector. These features represent the behaviours of the EEG signals, which are particularly significant for recognition and diagnosing purposes. In this paper, the eleven statistical features from each segment of EEG channel data are extracted as the valuable parameters for the representation of the characteristics of the original EEG signals which are mean (*X*_Mean_), median (*X*_Me_), mode (*X*_Mo_), standard deviation (*X*_SD_), first quartile (*X*_*Q*1_), third quartile (*X*_*Q*3_), inter-quartile range (*X*_IQR_), skewness (*X*_*β*1_), kurtosis (*X*_*β*2_), minimum (*X*_Min_), and maximum (*X*_Max_). It is noted that these features are the most representative values to describe the original EEG signal in each segment. The feature set is denoted by {*X*_Mean_, *X*_Me_, *X*_Mo_, *X*_*Q*1_, *X*_*Q*3_, *X*_IQR_, *X*_SD_, *X*_*β*1_,_*β*2_, *X*_Min_, *X*_Max_}. The accumulations of all obtained features from all segments of all classes are employed as the input for the three different classifiers.

### Detection

In this paper, three classifiers such as LMT, MLR, and SVM are used to evaluate the performance of the OAT feature set. The reason for choosing these classifiers for this study is its simplicity and effectiveness in implementation. They are also very powerful and fastest learning algorithm that examines all its training input for classification in this area. The following sections provide a brief idea about the classification methods that are used in this research.

#### Logistic model trees (LMT)

LMT have been shown to be very accurate and compact classifiers [13]. LMT are born out of the idea of combining two complementary classification schemes: linear logistic regression and tree induction. It has been shown that LMT perform competitively with other state-of-the-art classifiers such as boosted decision trees while being easier to interpret [13]. For the details of the LMT induction algorithm, the reader should consult [13].

#### Multinomial logistic regression with a ridge estimator (MLR)

MLR has become increasingly popular with the easy availability of appropriate computer routines. Ridge estimators are used in MLR to improve the parameter estimates and to diminish the error made by further prediction when maximum-likelihood estimators (MLE) are non-unique and infinite to fit data. When the number of explanatory variables is relatively large and or when the explanatory variables are highly correlated, the estimates of parameters are unstable, which means they are not uniquely defined (some are infinite) and/or the maximum of log likelihood is achieved at 0 [14, 15]. In this situation, ridge estimators are used to generate finiteness and uniqueness of MLE to overcome such problems. For the details of the MLR induction algorithm, the reader should consult [14, 15].

#### Support vector machine (SVM)

SVM is the most popular machines learning tool that can classify data separated by non-linear and linear boundaries, originated from Vapnik’s statistical learning theory [16].The main concepts of the SVM are to first transform input data into a higher dimensional space and then construct an optimal separating hyper plane (OSH) between the two classes in the transformed space [17, 18]. Those data vectors nearest to the constructed line in the transformed space are called the support vectors that contain valuable information regarding the OSH. SVM is an approximate implementation of the “method of structural risk minimization” aiming to attend low probability of generalization error. In most real-life problems (including our problem), the data are not linearly separable. In order to solve non-linear problems, SVMs use a kernel function [17, 18], which allows better fitting of the hyperplane to more general datasets. In more recent times, SVMs have been extended to solve multiclass-classification problems. One frequently used method in practice is to use a set of pairwise classifiers, based on one-against-one decomposition [18]. For the details of the SVM induction algorithm, the reader should consult [16–18].

## Experimental design

### Training and testing: cross cvalidation

There are many choices of how to divide the data into training and test sets [21]. In order to reduce the bias of training and test data, we propose employing *k*-fold cross-validation technique [21–24] considering *k* = 10. This technique is implemented to create the training set and testing set for evaluation. Generally, with *k*-fold cross validation, feature vector set is divided into *k* subsets of (approximately) equal size. The proposed classifiers are trained and tested *k* times, in which one of the subsets from training is left out each time and tested on the omitted subset. Each time, one of the subsets (folds) is used as a test set and the other *k−*1 subsets (folds) are put together to form a training set. Then, the average accuracy across all *k* trials is computed for consideration.

### Performance evaluation of classification schemes

Criteria for evaluating the performance of a classifier are an important part in its design. In this paper, we assess the performance of the proposed classifiers through most of the criteria that are usually used in biomedical research such as sensitivity, specificity, precision, F-measure, ROC, and total classification accuracy. These criteria allow estimating the behaviour of the classifiers on the extracted feature data. The evaluation measure most used in practice is the accuracy rate which evaluates effectiveness of the classifier by its percentage of correct prediction. More detailed descriptions of these evaluation criteria are discussed in [5].

## Data, experimental results, and discussions

We used the EEG time series database [19] which is publically available and is considered as a benchmark of testing classification techniques. The detailed descriptions of the dataset are discussed by Andrzejak et al. [20]. To validate the effectiveness of the proposed approach, we examine the scheme on the epileptic EEG database as discussed in Sect. 4.1. The experimental results are carried out in MATLAB (version 7.14, R2012a). We experimented three classification algorithms: LMT, MLR with a ridge estimator, and SVM implemented in WEKA machine learning toolkit [25]. LibSVM (version 3.2) [26] is used for the SVM classification in WEKA. In all of these cases, we consider the parameter values that have been used in WEKA default parameters settings.

According to our framework as discussed in Sect. 3, we divide each of the three classes (healthy, seizure-free, and seizure) into four parts (*k* = 4). As every channel of a class contains 4097 data points of 23.6 s, in each class, the sizes of each of the first three parts 1024 and the size of the fourth part are 1025, and each segment contains the data for 5.9 s. Then, we select a sample from each of the four parts in every class using the OAT scheme as discussed in Sect. 3. For this scheme, first determine the number of items need to be selected from each class, then determine the number of items from each and every part depending on the variability of the observations in that part. The required sample size under OAT scheme is given in Table 1.

**Table 1:** Sample sizes by the OAT scheme from each segment of each class

Different classes	Data sets	OAT procedure					Combined OAT sample
		Seg_1_	Seg_2_	Seg_3_	Seg_4_	Total OAT	
Healthy set	Set A	797	822	837	832	3288	6576
	Set B	815	840	805	828	3288	
Seizure-free set	Set C	839	841	780	828	3288	6576
	Set D	828	833	788	839	3288	
Seizure set	Set E	833	844	815	796	3288	3288

After selection of the samples from each of the four parts of each dataset by the OAT procedure, we combine all four samples of a dataset in a total set called “Total OAT” of that set. In this study, finally we combine the “Total OAT” of set A and set B denoted as the combined OAT sample for healthy set and for set C and set D denoted as the combined OAT sample of seizure-free set. Then we extract eleven features separately from the “Combined OAT sample” set of each class (healthy, seizure-free, and seizure) to represent the distribution pattern of that class.

Each set of A and B consists of 100 single channel EEG signals, thus the size of feature vector for the healthy class is 100 × 11 in the OAT schemes. Similarly, the size of feature vector for both the seizure-free class and the seizure class is 100 × 11. Thus, the size of whole feature vector for all three classes (health, seizure-free, and seizure) is 300 × 11. In this study, 10-fold cross-validation process is employed to generate training set and testing set for performance evaluation of the proposed algorithms. In each of the 10 iterations, the training set holds 250 × 11 data point, while the testing set contains 50 × 11 data point. Here the training set is used to train the classifier and the testing set is used to evaluate the accuracy and the effectiveness of the classifiers for the detection of the multiclass EEG data. In each classification system (LMT, MLR with a ridge estimator, and SVM), the training set is fed into the three different classifiers as the input to train the classifier and the performances are assessed with the testing test. It should be noted that in order to determine the consistency of the approach, each and every experiment is repeated twenty times and the average values of different performance parameters are reported.

**Fig. 2:**
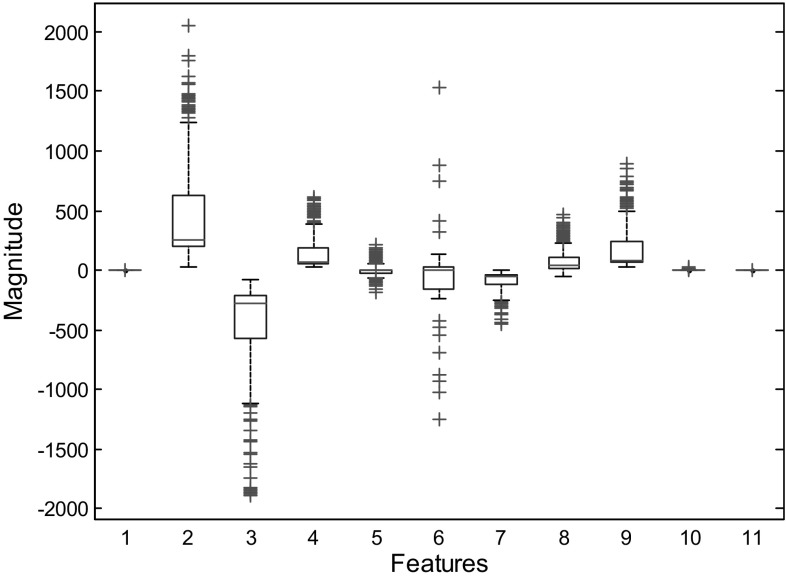
Box plot of obtained eleven features in the whole feature set to show their distribution

To illustrate the distribution of different features (11 features) of the OAT scheme, Fig. 2 presents a side by side box plot of different features. For example, the first box plot represents the distribution of the mean, the second box plot represents the distribution of the median, and so on. It should be noted that the features are plotted combining all the healthy, seizure-free, and seizure classes. As we can see from Fig. 2, the statistical features of mean, minimum, and the maximum obtained from the OAT scheme are almost same (constant) and so there is no well-designed distributions. For other features, the distributions are asymmetric (either positively skewed or negatively skewed) with some outliers. The outliers are expected as the features come from all classes of healthy, seizure-free, and seizure set.

As said before, to explore the performance of the OAT features, we tested three machine leaning methods: LRT, MLR with a ridge estimator, and SVM for detection of epileptic seizure EEG signals (healthy, seizure-free, and seizure). Table 2 reports the detection performance for the LRT classifier for the OAT features. This table provides different performance parameter (sensitivity, specificity, precision, F-measure, and ROC) values for each of the three classes (healthy, seizure-free, and seizure) in addition to the overall performance. As shown in Table 2, all of the performances indicators demonstrate excellent detection of three categories (healthy, seizure-free, and seizure) EEG signals by the LMT classifier with OAT scheme. In this case, all of the measurements of sensitivity, specificity, precision, F-measure, and ROC for seizure class are 99, 99, 98.5, 98.5, and 99.4 %, respectively The total classification accuracy is 95.33 %, and the other performance parameters (sensitivity, specificity, precision, F-measure, and ROC) are at least 92 % for both healthy and seizure-free classes.

**Table 2:** Performances of the LMT classifier on the OAT scheme

Class	Sensitivity (%)	Specificity (%)	Precision (%)	F-measure (%)	ROC	Total accuracy (%)
Healthy	95.0	97.0	94.10	94.50	0.993	95.33
Seizure-fee	92.0	97.0	93.90	92.90	0.978	
Seizure	99.0	99.0	98.0	98.50	0.994	
Overall	95.30	97.70	95.30	95.30	0.988	

Tables 3 and 4 display the classification results of the MLR and SVM classifiers under the OAT approach. As shown in Table 3, the overall classification accuracy is 82.67 % for the OAT scheme based on MLR approach. In this table, the sensitivity and specificity for seizure class are 98 and 100 %, whereas these performances for healthy class are 80.0 and 85.0 % and for seizure-free class are 70.0 and 89.0 %, respectively.

**Table 3:** Performances of the MLR classifier on the OAT scheme

Class	Sensitivity (%)	Specificity (%)	Precision (%)	F-measure (%)	ROC	Total accuracy (%)
Healthy	80.0	85.0	72.70	76.20	0.901	82.67
Seizure-fee	70.0	89.0	76.10	72.90	0.894	
Seizure	98.0	100.0	100.0	99.0	0.999	
Overall	82.70	91.30	82.90	82.70	0.932

**Table 4:** Performances of the SVM classifier on the OAT scheme

Class	Sensitivity (%)	Specificity (%)	Precision (%)	F-measure (%)	ROC	Total accuracy (%)
Healthy	4.0	100	100	7.70	0.52	36.0
Seizure-fee	4.0	100	100	7.70	0.52	
Seizure	100	4.0	34.2	51.0	0.52	
Overall	36.0	68.0	78.10	22.10	0.52	

We can also see in Table 4 that the OAT technique achieves only 36.0 % of the overall classification accuracy for the SVM classifier. This may be due to that fact that, under the OAT approach, the statistical features do not represent the whole EEG signals for the SVM classifier. According to the classification results as displayed in Tables 2, 3 and 4, it is obvious that the OAT scheme is a very reasonable way for achieving representative information from various categories EEG signals and the LMT classifier is the best suited with the OAT-based features for detecting multi-category EEG signals.

**Fig. 3:**
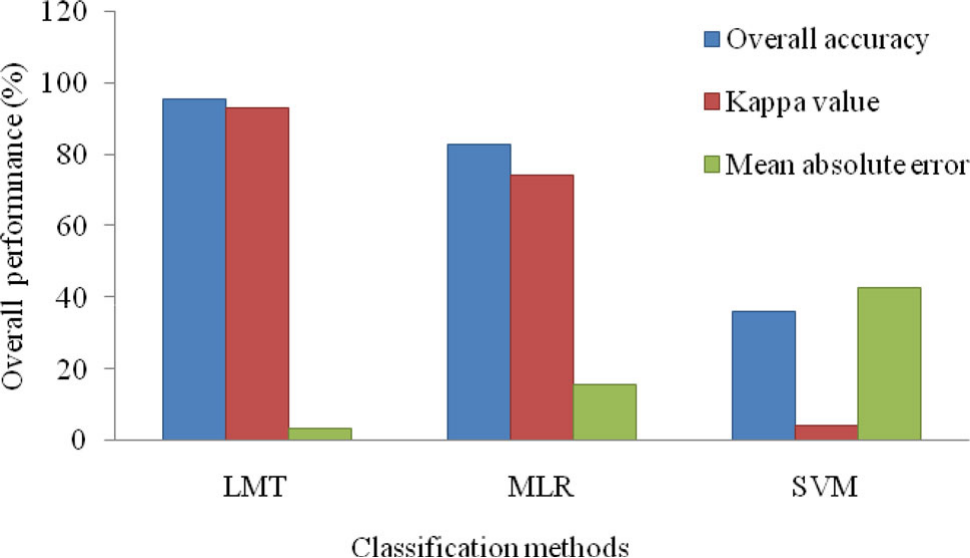
Overall performance comparison for the LMT, MLR, and SVM classifier on OAT scheme

Figure 3 displays a stacked bar diagram showing the overall classification accuracy, kappa value, and the mean absolute error. The highest overall accuracy and kappa values represent the paramount performance, whereas the highest mean absolute error represents worst performance. As we can see from Fig. 3, the highest overall accuracy and kappa value and the lowest mean absolute are achieved for the LMT classifier. The SVM classifier has a worst performance in respect of all the performance parameters of overall accuracy, kappa value, and the mean absolute error. On the other hand, the MLR classifier has a moderate performance. Thus, the statistical features obtained from the OAT scheme can be used as an input vector and the LMT can be used as a detection technique for detecting epileptic seizure EEG signals.

## Conclusion

Accurate and perfect detection of epileptic seizure EEG signals is a complicated problem, requiring the analysis of large sets of EEG data. This paper proposes a structure based on sampling and machine learning approach to detect multi-category EEG signals. The OAT scheme are employed to select representative samples from different parts of multi-category EEG signals. We tested this methodology on benchmark epileptic EEG database. To determine the consistency of the approach, each and every experiment is repeated twenty times and the average values of the performances are reported. The experimental results show that the features obtained from the OAT well represent the epileptic seizure EEG signals and achieve the consistent detection rates in terms of overall classification accuracy, class specific sensitivity, specificity, and the other detection parameters with the LMT classifier. Thus, the OAT can be used as a perfect scheme for feature extractions, while the LMT can be considered as an optimum choice with it for the detection of multi-category EEG signals. The proposed method may be applied for analysis and classification of other non-stationary biomedical signals.

## References

[1] Siuly S, Li Y (2014). A novel statistical algorithm for multiclass EEG signal classification. Eng Appl Artif Intell.

[2] Eiedermeyer E, Silva F. L. da (2005) Electroencephalography: basic principles, clinical applications, and related fields. Lippincott Williams and Wilkins, Philadelphia, Pa, USA, 5th edition

[3] Bajaj V, Pachori RB (2012). Classification of seizure and nonseizure EEG signals using empirical mode decomposition. IEEE Trans Inf Technol Biomed.

[4] Kutlu Y, Kuntalp M, Kuntalp D (2009). Optimizing the performance of an MLP classifier for the automatic detection of epileptic spikes. Expert Syst Appl.

[5] Siuly S, Kabir E, Wang H, Zhang Y (2015). Exploring sampling in the detection of multi-category EEG signals. Comput Math Methods Med.

[6] Hanbay D (2009). An expert system based on least square support vector machines for diagnosis of the valvular heart disease. Expert Syst Appl.

[7] Shen CP, Chen CC, Hsieh SL, Chen WH, Chen JM, Chen CM, Lai F, Chiu MJ (2013). High-performance seizure detection system using a wavelet-approximate entropy-fSVM cascade with clinical validation. Clin EEG Neurosci.

[8] Acharjee PP, Shahnaz C (2012) Multiclass epileptic seizure classification using time-frequency analysis of EEG signals. In: Proceedings of the 7th International Conference on Electrical and Computer Engineering -ICECE 12. pp. 260–263, Dhaka, Bangladesh

[9] Murugavel ASM, Ramakrishnan S, Balasamy K, Gopalakrishnan T (2011). Lyapunov features based EEG signal classification by multi-class SVM. Proceedings of the world congress on Information and Communication Technologies—WICT 11.

[10] Ubeyli ED (2009). Decision support systems for time-varying biomedical signals: EEG signals classification. Expert Syst Appl.

[11] Ubeyli ED (2008). Analysis of EEG signals by combining eigenvector methods and multiclass support vector machines. Comput Biol Med.

[12] Guler I, Ubeyli ED (2007). Multiclass support vector machines for EEG-signals classification. IEEE Trans Inf Technol Biomed.

[13] Landwehr N, Hall M, Frank E (2005). Logistic model trees. Mach Learn.

[14] Cessie SL, Van Houwelingen JC (1992). Ridge estimators in logistic regression. Appl Stat.

[15] Zahid FM, Tutz G (2013). Ridge estimation for multinomial logit models with symmetric side constraints. Comput Stat.

[16] Vapnik VN (2000). The nature of statistical learning theory.

[17] Begg RK, Palaniswami M, Owen B (2005). Support vector machines for automated gait classification. IEEE Trans Biomed Eng.

[18] Yin X, Ng BWH, Fischer BM, Ferguson B, Abbott D (2007). Support vector machine applications in terahertz pulsed signals feature sets. IEEE Sens J.

[19] EEG time series (2005), http://www.meb.uni-bonn.de/epileptologie/science/physik/eegdata.html

[20] Andrzejak RG, Lehnertz K, Mormann F, Rieke C, David P, and Elger CE (2001) Indications of nonlinear deterministic and finite-dimensional structures in time series of brain electrical activity: dependence on recording region and brain state. Phys Rev E 64 (6) 10.1103/PhysRevE.64.061907 11736210

[21] Chaovalitwongse WA, Fan YJ, Sachdeo RC (2007). On the time series *K*-nearest neighbor classification of abnormal brain activity. IEEE Trans Syst Man Cybern Part A.

[22] Efron B (1983). Estimating the error rate of a prediction rule: improvement on cross-validation. J Am Stat Assoc.

[23] Siuly S, Li Y (2012). Improving the separability of motor imagery EEG signals using a cross correlation-based least square support vector machine for brain-computer interface. IEEE Trans Neural Syst Rehabil Eng.

[24] Sengur A (2009). Multiclass least-squares support vector machines for analog modulation classification. Expert Syst Appl.

[25] Frank E, Hall M, Holmes G, Kirkby R, Pfahringer B, Witten IH, Trigg L (2010) Weka—a machine learning workbench for data mining. In Data Mining and Knowledge Discovery Handbook, pp. 1269–1277, Springer US, 2010

[26] Chang CC, Lin CJ (2011) LIBSVM: a library for support vector machines. ACM Transactions on Intelligent Systems and Technology

